# Mortality, morbidity, and predictors of death among amphetamine-type stimulant users − a longitudinal, nationwide register study

**DOI:** 10.1016/j.abrep.2024.100553

**Published:** 2024-05-14

**Authors:** A. Åhman, A. Karlsson, J. Berge, A. Håkansson

**Affiliations:** a Lund University, Faculty of Medicine, Department of Clinical Sciences Lund, Psychiatry, Lund, Sweden; b Faculty of Medicine, Department of Clinical Sciences Lund, BMC F12, Sölvegatan 19, 221 84 Lund, Sweden

**Keywords:** ATS, Amphetamine type-stimulants, Stimulants, Morbidity, Mortality, Causes of death

## Abstract

**Introduction:**

Use of amphetamine-type stimulants (ATS) contributes substantially to the global burden of disease. Large-scale follow-up studies of morbidity and mortality in ATS users are few. This study analysed morbidity, mortality, and potential predictors of all-cause mortality in a nationwide cohort of patients with ATS use disorder.

**Methods:**

Data was acquired from national Swedish registers. All Swedish residents 18 years or older, with a registered ATS use diagnosis in 2013–2014 were included (N = 5,018) and followed until December 31, 2017. Comorbid diagnoses and causes of death were assessed and potential predictors of all-cause mortality were examined through Cox regression.

**Results:**

Median age at inclusion was 36.6 years (interquartile range 27.4–––48.1) and 70.5 % were men. The crude mortality rate was 24.6 per 1,000 person-years. The adjusted all-cause standardized mortality ratio was 12.4 (95 % CI [11.34–13.55]). The most common cause of death was overdose (28.9 %). Multiple drug use (hazard ratio 1.39, 95 % CI [1.14–1.70], p = 0.004), anxiety (hazard ratio 1.39, 95 % CI [1.11–1.72], p = 0.014), viral hepatitis (hazard ratio 1.85, 95 % CI [1.50–2.29], p = 0.004), and liver disease (hazard ratio 2.41, 95 % CI [1.55–3.74], p = 0.004) were predictors of all-cause mortality.

**Conclusions:**

Multiple drug use, anxiety disorders, viral hepatitis and liver diseases were identified as risk factors for death. Our findings call for better screening, prevention, and treatment of somatic and psychiatric comorbidity among ATS users to reduce mortality.

## Highlights

• ATS users exhibit a significant psychiatric and somatic comorbidity.
• A high proportion have multiple drug use.
• Common causes of death include overdose, cardiovascular disease, and suicide.
• Multiple drug use, viral hepatitis, and liver disease are risk factors for death.

## 1 Introduction

Amphetamine-type stimulants (ATS) include amphetamine, methamphetamine, 3,4-methylenedioxy-methamphetamine (MDMA) and related substances as well as prescribed medications (e.g., lisdexamphetamine and methylphenidate). The use of ATS has increased in most countries in the world in the past decade and The United Nations estimate the number of past year amphetamine users in 2021 to be 36 million and of ecstasy 20 million (United Nations Office on Drugs and Crime, 2023).

Methamphetamine dominates the ATS seizures at a global level and amphetamine dominates in Western, Central and South-Eastern Europe as well as in the Middle East (United Nations Office on Drugs and Crime, 2023). As for Sweden, amphetamine is the dominating drug among people who inject drugs (Kåberg et al., 2017).

ATS use is associated with severe health effects both in the acute and in the chronic phase, both due to the acute drug toxicity and to the lifestyle that often follows an addiction. Possible acute and sub-acute effects include increased heart rate and elevated blood pressure, hyperthermia, insomnia, anxiety, and acute psychosis (Darke et al., 2008, Heal et al., 2013). People with amphetamine use disorder are at greater risk for depression, violence and suicidality (McKetin et al., 2019), as well as acute (Callaghan et al., 2018, Kaye et al., 2007) and chronic cardiovascular pathology (Darke et al., 2017, Jafari Giv, 2017). In addition to adverse physiological and psychological health effects, amphetamine users are at an elevated risk of dying of various causes such as, overdose, suicide, and accidents (Stockings et al., 2019).

Morbidity and mortality in the ATS-using population have previously been poorly researched (Singleton et al., 2009). However, the number of studies has increased in recent years (Stockings et al., 2019). Mortality rates among ATS users have been reported to be lower than among opioid users (Bartu et al., 2004, Wahren et al., 1997) but higher than in the general population (Åhman et al., 2018, Arendt et al., 2011). Stocking et al.'s *meta*-analysis from 2019, including 23 cohorts, showed a significant excess mortality, with a pooled all-cause standardized mortality ratio (SMR) of 6.8 (95 % CI [5.27–8.84]) (Stockings et al., 2019). To better understand the high mortality among ATS users, it is necessary to examine comorbid conditions. This is also important for study comparisons and was requested in the *meta*-analysis on ATS users' mortality (Stockings et al., 2019). Additionally, studies reporting specific causes of death with corresponding codes according to the 10th revision of the International Classification of diseases (ICD-10) are needed to facilitate comparisons between cohorts (Stockings et al., 2019). The two aims of this study were therefore to: (i) examine the morbidity, mortality, and causes of death within a national cohort of ATS users using diagnostic data according to ICD-10, and (ii) assess potential predictors of all-cause mortality within this population.

## 2 Materials and methods

### 2.1 Data sources

Data acquired from the Swedish National Patient Registry (NPR) were linked by personal identity number, to the Swedish Cause of Death Register (CDR). Both registers are held by the Swedish National Board of Health and Welfare. The NPR covers 99 % of all somatic and psychiatric hospital discharges and around 80 % of all hospital-based outpatient care and contains data on primary and secondary diagnoses according to the Swedish version of the ICD-10 (Ludvigsson et al., 2011).

The CDR covers almost all deaths in Sweden since 1952 and is based on all death certificates issued during a calendar year. According to Swedish legislation, all suspected unnatural deaths, including drug-related deaths, are routinely investigated through forensic autopsy (Brooke et al., 2017). B The forensic pathologist who performs the autopsy determines the cause of death according to ICD. Both the underlying cause of death and any number of contributing causes are included in the CDR (The Swedish National Board of Health and Welfare, 2022).

### 2.2 Study population and outcome variables

The study includes all Swedish residents 18 years or older with a registered ATS use diagnosis, defined as the ICD-10 diagnosis F15 (“Mental and behavioural disorders due to use of other stimulants, including caffeine”), i.e. all stimulants except for cocaine, either as a primary or a secondary diagnosis in the NPR at any time between January 1, 2013 and December 31, 2014.

Data on age at inclusion, gender and a selection of comorbid diagnoses registered up to two years prior the ATS diagnosis, were obtained from the NPR. The age variable was divided into four age categories: <30 years, 30–44 years, 45–59 years and > 59 years. The category < 30 years was used as reference. The comorbid diagnoses were other substance use disorders and psychiatric disorders and somatic conditions previously associated with ATS use (Gahrton et al., 2021, Hartzler et al., 2011, Kaye et al., 2007, McKetin et al., 2019). We also included self-harm and accidental poisoning. A complete list of included comorbid diagnoses and respective ICD-10 codes is presented in Supplementary Table S1 (Supplementary Table S1). Data on death dates and causes of deaths during the follow up was obtained from the CDR.

### 2.3 Statistical analysis

Cox regression was used to assess the association between comorbid diagnoses and death. The mortality event was first regressed against the comorbid diagnoses respectively, adjusted for age at inclusion and sex, and then adjusted for age, sex and all comorbid diagnoses. Time-at-risk was calculated from inclusion date (occurrence of an F15 diagnosis in the NPR between 2013–2014) and the time to death or to termination of follow-up on December 31, 2017, whatever occurred first. Results are presented as Hazard Ratios (HR) with 95 % confidence intervals (CI). P-values below the value of 0.05 were considered statistically significant. The proportionality assumption was verified by scaled Schoenfeld residuals (Kleinbaum & Klein, 2012). To restrain the false discovery rate to 5 % among the 21 Cox regression analyses in the multivariable model, the Benjamini Hochberg (BH) correction procedure was performed on the p-values from the analyses (Hochberg & Benjamini, 1990). We used R version 4.0.2 for data preparation. The statistical analysis was performed in SPSS version 29. Standardized mortality ratios (SMRs) (Kelsey & Gold, 2017) were calculated using age- (in five-year categories) and sex-specific mortality rates from the general Swedish population obtained from a national database (The Swedish National Board of Health and Welfare, 2024). To adjust for individuals aging into higher age categories and the associated increased mortality rates, an adjusted SMR was computed by allocating half of the observation time in each age category to the subsequent age category. Calculations were conducted using Microsoft Excel version 16.84, and 95 % CIs were computed using an online tool (Dean et al., 2013).

### 2.4 Ethics

The study was approved by the regional ethics board, Lund, Sweden (file number 2018/147). Due to the study design consent was not obtained.

## 3 Results

### 3.1 Sample characteristics

A total of 5,018 individuals (N = 5,018) aged 18 or older were diagnosed with an ATS use diagnosis in Sweden in 2013–2014. The follow-up time was a maximum of five years and median follow-up time (i.e. time at risk) was 4.1 years (interquartile range [IQR] 3.5–––4.6) with a total follow-up time for the entire cohort of 19654.7 person-years (py). The median age at inclusion was 36.6 years (IQR 27.4–––48.1). The largest age group was between 30–44 years, constituting 35.4 % of the cohort. A majority were men, 70.5 % (n = 3,540). The demographic data of the cohort are presented in Table 1.

**Table 1:** Descriptive data on the total study population (N = 5,018) and on the deceased. Study inclusion 2013–2014. Comorbid diagnosis data two years prior to study inclusion.

Variables	Total, N = 5,018		Deceased, N = 484	
Female sex, N (%)	1478	(29.5)	103	(21.3)

*Age groups at study start, N (%)*				
<30	1636	(32.6)	120	(24.8)
30–44	1778	(35.44)	137	(28.3)
45–59	1382	(27.5)	161	(33.3)
>59	222	(4.4)	66	(13.6)
Time in person-years observed, median (IQR)	4.1	(3.5–4.6)	1.6	(0.63–2.8)

*Substance use disorders, N (%)*				
Alcohol	1587	(31.6)	182	(37.6)
Opioids	720	(14.3)	107	(22.1)
Cannabis	663	(13.2)	56	(11.6)
Sedatives	681	(13.6)	99	(20.5)
Cocaine	136	(2.7)	14	(2.9)
Multiple drug use	2242	(44.7)	278	(57.4)

*Psychiatric diagnoses, N (%)*				
Depression	792	(15.8)	80	(16.5)
Anxiety	1098	(21.9)	133	(27.5)
Psychosis	742	(14.8)	80	(16.5)
ADHD/ADD	1440	(28.7)	136	(28.1)

*External causes of morbidity, N (%)*				
Self-harm	616	(12.3)	81	(16.7)
Accidental poisoning	236	(4.7)	38	(7.9)

*Somatic diagnoses, N (%)*				
Hypertension	154	(3.1)	36	(7.4)
Ischemia	60	(1.2)	23	(4.8)
HIV	51	(1.0)	12	(2.5)
Viral hepatitis	967	(19.3)	184	(38.0)
Liver disease	61	(1.2)	23	(4.8)

Abbreviations: attention deficit hyperactivity disorder (ADHD), attention deficit disorder (ADD), human immunodeficiency virus (HIV).

### 3.2 Comorbidity

Alcohol use disorder was the most frequent (31.6 %) concomitant single substance use disorder in the cohort, followed by opioid use disorder (14.3 %) (Table 1). Multiple drug use disorder was present in 44.7 % of the cohort. Moreover, 28.7 % had attention deficit hyperactivity disorder (ADHD) or attention deficit disorder (ADD), 21.9 % of the study participants suffered from anxiety disorder, and 15.8 % from a depressive disorder. Furthermore, 12.3 % of the cohort had a history of intentional self-harm and 4.7 % had overdosed.

Viral hepatitis, the most common somatic disorder, had the largest discrepancy between representation in the total cohort 19.3 % and among the deceased (38.0 %) (Table 1). Conversely, cannabis use disorder and ADHD/ADD were more prevalent among the living than the deceased.

### 3.3 Mortality and causes of death

During follow up, a total of 484 participants deceased (Table 1). The crude mortality rate (CMR) was 24.6 per 1,000 py (95 % CI 22.5–26.9). The all-cause SMR was 15.6 (95 % CI [14.27–17.05]) and adjusted all-cause SMR was 12.4 (95 % CI [11.34–13.55]). A total of 70.5 % of the deceased were men. The median age at death was 43.0 years (IQR 30.0–55.7), ranging from 18-93 years. The most frequent cause of death among the 484 deceased was accidental drug poisoning (28.9 %) (Table 2). Death due to diseases of the circulatory system were reported for 13.8 %, followed by deaths due to intentional self-harm (12.4 %) and poisonings with undetermined event (10.7 %). External causes of mortality as a group accounted for most of the deaths at 59.1 %.

### 3.4 Predictors of all-cause mortality

Age at inclusion above 59 years was, in comparison to age at inclusion < 30 years, a predictor of all-cause mortality (HR 3.51, 95 % CI [2.52–4.89], p = 0.004). Multiple drug use disorder (HR 1.39, 95 % CI [1.14–1.70], p = 0.004), anxiety disorder (HR 1.39, 95 % CI [1.11–1.72], p = 0.014), viral hepatitis (HR 1.85, 95 % CI [1.50–2.29], p = 0.004), and liver disease (HR 2.41, 95 % CI [1.55–3.74], p = 0.004) were also predictors of all-cause mortality. Female sex was negatively associated with death (HR 0.65, 95 % CI [0.52–0.82], p = 0.004).

## 4 Discussion

This study presents a longitudinal and broad insight of a nationwide ATS-using population (N = 5,018) regarding morbidity, mortality, and potential predictors of all-cause mortality. We found the typical ATS user to be male (70.5 %) and relatively young (36.6 years [IQR 27.4–––48.1]) (Table 1) in agreement with previous research (Åhman et al., 2018, Darke et al., 2017, Ericsson et al., 2014, Kuo et al., 2010).

### 4.1 Substance use disorders

In this study, 44.7 % of ATS users received a multiple drug use disorder diagnosis (Table 1). Medical practitioners may differ in their use of this diagnosis, using it alone or combined with individual substance use codes (Walderhaug et al., 2019). Multiple drug use is well-documented among ATS users (Butterworth et al., 2018, Darke et al., 2006, Timko et al., 2018). A history of injecting both heroin and methamphetamine has been shown to be associated with a 2.8 fold increase in reported overdose in the past 12 months compared to only injecting heroin (Al-Tayyib et al., 2017). Another study found injection of heroin mixed with methamphetamine in the past 3 months to be an independent predictor of overdose (Ochoa et al., 2005). This is in line with accidental poisoning being the most common cause of death in the cohort (28.9 %) (Table 2). Problematic alcohol use is known to be common among ATS users (Egan et al., 2012, Farrell et al., 2019, Gahrton et al., 2021, Hartzler et al., 2011), also supported in this study with 31.6 % of the participants having an alcohol use disorder diagnosis (Table 1).

**Table 2:** Underlying causes of death among 484 deceased amphetamine-type stimulant users.

ICD 10^a^	Diagnostic category	n	%	Number of deaths per 10,000 person-years
*Natural causes of death (n = 198, 40.9 %)*				
A00-B99	Certain infectious and parasitic diseases^b^	19	3.9	9.7
C00-D48	Neoplasms^c^	43	8.9	21.9
E00-E90	Endocrine, nutritional and metabolic diseases	10	2.1	5.1
F00-F99	Mental and behavioural disorders^d^	13	2.7	6.6
G00-G99	Diseases of the nervous system	3	0.6	1.5
I00-I99	Diseases of the circulatory system^e^	67	13.8	34.1
J00-J99	Diseases of the respiratory system	18	3.7	9.2
*K*00-K93	Diseases of the digestive system	12	2.5	6.1
M00-M99	Diseases of the musculoskeletal system and connective tissue	1	0.2	0.5
N00-N99	Diseases of the genitourinary system	2	0.4	1.0
R00-R99	Symptoms, signs and abnormal clinical and laboratory findings, not elsewhere classified^f^	10	2.1	5.1

*Unnatural causes of death (n = 286, 59.1 %)*				
V01-X59	Accidents (X40-X49 excluded)	22	4.5	11.2
X40-X49	Accidental poisoning by and exposure to noxious substances	140	28.9	71.2
X60-X84	Intentional self-harm	60	12.4	30.5
X85-Y09	Assault	1	0.2	0.5
Y10-Y34	Event of undetermined intent (Y11-Y14 excluded)	10	2.1	5.1
Y11- Y14	Poisoning, event of undetermined intent	52	10.7	26.5
Y35-Y36	Legal intervention and operations of war	1	0.2	0.5
Total		484	100.0	

a International Classification of Diseases 10th Revision

b Chronic viral hepatitis B (n = 1), chronic viral hepatitis C (n = 11), human immunodeficiency virus [HIV] disease (n = 1).

c Malignant neoplasms of the liver and intrahepatic bile ducts (n = 15), lung cancer (n = 9).

d Substance use disorder (n = 11).

e Ischemic heart diseases (n = 34), cerebrovascular diseases (n = 9).

### 4.2 Psychiatric comorbidity

The most frequent psychiatric comorbidity, aside from substance use disorders, was ADHD/ADD, found in 28.7 % of cases (Table 1). This is consistent with previous findings that ADHD/ADD diagnosis is frequently occurring among substance users (van Emmerik-van Oortmerssen et al., 2012). Anxiety was diagnosed in 21.9 % of the cohort, while depression was diagnosed in 15.8 % (Table 1), exceeding the estimated one-year prevalence in the general population, which is approximately 14 % for anxiety and 7 % for depression (Wittchen et al., 2011). Frequent amphetamine use is linked to greater likelihood of anxiety (Butterworth et al., 2018). However, a systematic *meta*-analysis from 2019 (McKetin et al., 2019), showed no significant association between having an amphetamine use disorder and anxiety, although the number of studies was limited. The connection between ATS use and depression is more documented (McKetin et al., 2019) and depressive symptoms are common in people seeking treatment for methamphetamine use (McKetin et al., 2011).

### 4.3 Somatic comorbidity

Viral hepatitis was present in 19.3 % of the cohort (Table 1), likely related to injection drug use since hepatitis C virus (HCV) infection is a common injecting-related diseases and amphetamine being the most common drug among Swedish injection users (Kåberg et al., 2017). Although HIV is also associated with injection drug use, Sweden has a low HIV prevalence (Public Health Agency of Sweden, 2023), with only 1 % of the cohort diagnosed.

### 4.4 Mortality and causes of death

The CMR of this cohort was 24.6 per 1,000 py (95 % CI 22.5–26.9). In the *meta*-analysis of mortality among people with regular or problematic use of amphetamines (Stockings et al., 2019), the pooled all-cause CMR for 23 cohorts of amphetamine users was 11.4 per 1,000 py. The CMR was highest in studies from Southeast Asia (20.2 per 1,000 py) and Western Europe (11.7 per 1,000 py). CMR estimates were significantly lower for studies using national-level data than studies using subnational or city-level recruitment (Stockings et al., 2019). The adjusted all-cause SMR in our study was notably elevated at 12.4 (95 % CI [11.34–13.55]). This figure can be compared with the pooled all-cause SMR of 23 studies reported by Stocking et al., which was 6.8 (95 % CI 5.27–8.84) (Stockings et al., 2019). Our study’s comparable high CMR and SMR could be partly due to not including exclusively individuals receiving treatment for their substance use (Bartu et al., 2004). Another possible explanation for higher figures is that polysubstance use could be more prevalent in this study, which could contribute to increased mortality.

A majority (59.1 %) of the 484 deceased had an unnatural cause of death (Table 2). Consistent with earlier studies (Calcaterra and Binswanger, 2013, Darke et al., 2017, Kaye et al., 2008), the most common cause of death was accidental poisoning (28.9 %). A Swedish study based on a regional syringe exchange cohort of amphetamine users (Åhman et al., 2018), reported that 7 % of the participants deceased due to accidental poisoning. In another Swedish study (Ericsson et al., 2014), focusing on amphetamine users within the criminal justice system, the proportion of death from accidental poisoning was notably higher at 18 %. Poisonings of undetermined intent accounted for 10.7 % of fatalities. When added to accidental poisonings, poisonings as a whole made up 39.6 % of cohort deaths. The proportion of death due to suicide in this cohort was 12.4 % (Table 2). Previous studies have reported percentages in the range 6–32 % (Åhman et al., 2018, Darke et al., 2017, Ericsson et al., 2014, Kaye et al., 2008, Kuo et al., 2010, Stenbacka et al., 2010).

Natural causes accounted for 40.9 % of the deaths (Table 2), which is higher compared to Darke et al. – 22.3 % (Darke, Kaye, et al., 2017). This study explores all-cause mortality among ATS users, irrespective of whether ATS use was cited as a cause of death. Additionally, it includes not only methamphetamine users but also users of other ATS. Circulatory system diseases caused 13.8 % of all deaths, ranking as the most common natural cause and the second most common overall (Table 2), aligning with the documented link between ATS use and acute and chronic cardiovascular disease (Callaghan et al., 2018, Darke et al., 2017, Kaye et al., 2007, Turner et al., 2018). Similar numbers have been reported, ranging between 10–19 % (Åhman et al., 2018, Darke et al., 2017, Kuo et al., 2010, Stenbacka et al., 2010). Chronic cardiovascular diseases progress gradually. Extending the 4.1-year follow-up might reveal more cardiovascular deaths. Focusing solely on ATS dependence could potentially increase the proportion of cardiovascular deaths.

### 4.5 Predictors of all-cause mortality

Age above 59 years was found to be risk factor for death compared to age < 30 years, in line with previous research (Åhman et al., 2018, Darke et al., 2006). Old age independently increases the risk of death, and old age in this population also could represent a longer period of ATS use. It is not possible with the present study design to distinguish these two explanatory factors from one another. Female sex was found to be a protective factor, consistent with existing evidence (Åhman et al., 2018, Darke et al., 2006).

Multiple drug use disorder and anxiety disorder were the only psychiatric comorbidities that remained significant predictors of all-cause mortality in the multivariable adjusted analysis (Table 3). ATS together with opioids, sedatives or alcohol increases the risk of overdose (Al-Tayyib et al., 2017, Darke et al., 2006) − which was also the most common cause of death in this cohort (Table 2). However, this finding in this national material highlights a need to address and treat the multiple drug use in the ATS using population. This may include similar interventions as in opioid use disorder, such as harm reduction measures, overdose prevention counselling and naloxone treatment. In the adjusted multivariate analysis, opioid use disorder did not emerge as a predictor of mortality. It can be assumed that a diagnosis of multiple drug use disorder is such a robust risk factor for all-cause mortality, that it surpasses other supposed risk factors, such as opioid use disorder.

**Table 3:** Associations between comorbidity and all-cause mortality among 5,018 amphetamine type-stimulant users. Cox regression. Time-at-risk calculated from date of inclusion (2013–2014) to event (all cause death) or end of follow up December 31, 2017.

Variables	Obs. Time (py)	Events (n)	uHR^a^	95 % CI	aHR^b^	95 % CI	P-value^c^	aP-value^d^
Female sex	5,918	103	0.67	0.53–0.83	0.65	0.52–0.82	**<0.001**	**0.004**

*Age groups at inclusion*								
<30	6,405	120	−	−	−	−	−	−
30–44	7,066	137	1.00	0.79–1.28	0.93	0.72–1.19	0.554	0.727
45–59	5,412	161	1.53	1.21–1.95	1.31	1.02–1.69	0.038	0.098
>59	772	66	4.28	3.16–5.78	3.51	2.52–4.89	**<0.001**	**0.004**

*Substance use disorders*								
Alcohol	6,215	182	1.28	1.06–1.54	1.00	0.82–1.21	0.978	0.978
Opioids	2,707	107	1.91	1.54–2.38	1.28	1.01–1.63	0.042	0.098
Cannabis	2,640	56	0.93	0.70–1.23	0.88	0.66–1.18	0.392	0.557
Sedatives	2,579	99	1.87	1.49–2.33	1.25	0.97–1.60	0.079	0.166
Cocaine	527	14	1.26	0.74–2.14	1.05	0.61–1.80	0.866	0.949
Multiple drug use	8,615	278	1.86	1.55–2.23	1.39	1.14–1.70	**0.001**	**0.004**

*Psychiatric diagnoses*								
Depression	3,095	80	1.14	0.89–1.45	0.96	0.75–1.24	0.77	0.898
Anxiety	4,181	133	1.65	1.35–2.03	1.39	1.11–1.72	**0.004**	**0.014**
Psychosis	2,941	80	1.11	0.90–1.45	1.04	0.82–1.33	0.738	0.898
ADHD/ADD	5,768	136	1.03	0.85–1.26	0.92	0.75–1.12	0.398	0.557

*External causes of morbidity*								
Self-harm	2,355	81	1.71	1.14–2.18	1.02	0.76–1.36	0.904	0.949
Accidental poisoning	887	38	1.96	1.41–2.73	1.22	0.83–1.79	0.312	0.504

*Somatic diagnoses*								
Hypertension	559	36	1.83	1.29–2.60	1.22	0.83–1.79	0.31	0.504
Ischemia	195	23	2.68	1.73–4.15	1.67	1.04–2.68	0.034	0.098
HIV	192	12	2.17	1.22–3.85	1.52	0.84–2.74	0.164	0.313
Viral hepatitis	3,579	184	2.47	2.04–2.98	1.85	1.50–2.29	**<0.001**	**0.004**
Liver disease	189	23	3.95	2.59–6.03	2.41	1.55–3.74	**<0.001**	**0.004**

Abbreviations: observation time (Obs. Time), person-years (py), Hazard Ratios (HR), conference intervals (CI), attention deficit hyperactivity disorder (ADHD), attention deficit disorder (ADD), human immunodeficiency virus (HIV).

a Adjusted for age groups and sex.

b All variables included in the analysis.

c P-values for the aHR analysis.

d Benjamini–Hochberg adjusted p-values using all 21p-values.

Somewhat surprisingly, anxiety disorder was found to predict death. Some of the potential acute effects of ATS use include panic and anxiety (Darke et al., 2008) and withdrawal from heavy ATS use has shown potential to initiate or worsen anxiety problems (McGregor et al., 2005). Anxiety disorder could thus in part reflect a more serious substance use in this cohort. It is also conceivable that anxiety disorders might lead to an increased prescription of benzodiazepines, potentially contributing to an increased risk of overdose. Regardless, our results provide reasons to pay attention to anxiety problems among ATS users, and to investigate any underlying or associated problems. Further studies, controlling for several confounding factors and follows the conditions over time, are warranted.

Viral hepatitis and liver disease were the assessed somatic co-morbidities that predicted death in the cohort, also showing the highest HR, 1.85 and 2.41 respectively (Table 3). Viral hepatitis is common among people who inject drugs (Kåberg et al., 2017). Untreated HCV may lead to end stage liver cirrhosis and hepatic failure (Freeman et al., 2001) and the proportion of treated HCV infected ATS users in Sweden have been reported to be low (Gahrton et al., 2021). Compared to opioid users, ATS users may have less contact with healthcare providers due to the absence of substitution treatment, potentially limiting access to antiviral treatment. Problematic alcohol use among ATS users may also be a contributing factor (Egan et al., 2012, Farrell et al., 2019, Gahrton et al., 2021, Hartzler et al., 2011). This could additionally contribute to liver cirrhosis development and impair the outcome of HCV treatment. Our findings implicate that screening and treatment of hepatitis infection and liver diseases as well as associated conditions such as alcohol use disorders, may reduce mortality in ATS users.

### 4.6 Limitations

Several limitations should be noted. While Sweden's national registers have high coverage, the NPR lacks primary care data, meaning ATS users solely followed by primary care units are not represented. The same applies to those with no contact with authorities or only with social services, which also handle addictive disorder treatment in Sweden. However, many of these patients likely visit specialized healthcare within two years and are thus included here. Additionally, this register-based study reflects comorbidities up to two years before the ATS diagnosis, excluding earlier diagnoses. Information on substance use extent, frequency, and administration route is lacking. The broad ATS substance category (F15 diagnosis) prevents discrimination between specific substances. Due to the observational design, causal mechanisms cannot be concluded. While age and sex were adjusted for in Cox regression, other potential confounders like socioeconomic data were not included. Future studies should incorporate such data and compare with other substance use disorders, like opioids, to better understand ATS-related risks. Longer observation times would enhance statistical power, especially given limited case numbers in numerous cause-of-death categories.

## 5 Conclusion

Swedish ATS users have high rates of both psychiatric and somatic comorbidity and major cause of death are accidental drug overdosing. Multiple drug use, anxiety disorders, viral hepatitis and liver diseases are risk factors for death in this group of substance users. These findings suggest a need for better screening and treatment of multiple drug use among individuals with ATS use disorder to reduce mortality. Efforts to increase hepatitis treatment, as well as prevention and treatment of liver diseases among ATS users, may also reduce mortality in this group.

## CRediT authorship contribution statement

**A. Åhman:** Writing – review & editing, Writing – original draft, Validation, Project administration, Methodology, Investigation, Formal analysis, Conceptualization. **A. Karlsson:** Writing – review & editing, Validation, Methodology, Conceptualization. **J. Berge:** Writing – review & editing, Validation, Supervision, Project administration, Methodology, Data curation, Conceptualization. **A. Håkansson:** Writing – review & editing, Validation, Supervision, Project administration, Methodology, Conceptualization.

## Declaration of competing interest

The authors declare that they have no known competing financial interests or personal relationships that could have appeared to influence the work reported in this paper.

## Data Availability

The authors do not have permission to share data.
