# Supplementary material for: Mortality, morbidity, and predictors of death among amphetamine-type stimulant users − a longitudinal, nationwide register study
Source: Addict Behav Rep. 2024 May 14;19:100553. doi: 10.1016/j.abrep.2024.100553 (PMC11127464; doi:10.1016/j.abrep.2024.100553)
Supplement: Supplementary Data 1 [file mmc1.docx]

| **Supplementary table 1**  Categories of comorbid diagnoses and included ICD-10 codes. | | | |
| --- | --- | --- | --- |
| **Diagnostic category** | | | **ICD 10^a^** |
|  |  |  |  |
| *Substance use disorders* | | |  |
| Alcohol use disorder | | | F10 |
| Opioid use disorder | | | F11 |
| Cannabis use disorder | | | F12 |
| Sedatives use disorder | | | F13 |
| Cocaine use disorder | | | F14 |
| Multiple drug use disorder | | | F19 |
|  | | |  |
| *Psychiatric diagnoses* | | |  |
| Depressive disorder | | | F32-F33 |
| Anxiety disorder | | | F41 |
| Psychotic disorder | | | F20-F29 |
| ADHD/ADD | | | F90 |
|  | | |  |
| *External causes of morbidity* | | |  |
| Self-harm | | | X60-X84 |
| Accidental poisoning | | | X40-X49 |
|  | | |  |
| *Somatic diagnoses* | | |  |
| Hypertension | | | I10-I15 |
| Ischemia | | | I20-I25 |
| HIV^b^ | | | B20-B24 |
| Viral hepatitis | | | B15-B19 |
| Liver disease | | | K70-K77 |
|  | | |  |
| Abbreviations: attention deficit hyperactivity disorder (ADHD), attention deficit disorder (ADD), human immunodeficiency virus (HIV)  ^a^ International Classification of Diseases 10th Revision | | | |
